# *Notes from the Field: *Opioid Overdose Deaths Before, During, and After an 11-Week COVID-19 Stay-at-Home Order — Cook County, Illinois, January 1, 2018–October 6, 2020

**DOI:** 10.15585/mmwr.mm7010a3

**Published:** 2021-03-12

**Authors:** Maryann Mason, Sarah B. Welch, Ponni Arunkumar, Lori Ann Post, Joseph M. Feinglass

**Affiliations:** ^1^Feinberg School of Medicine, Northwestern University, Chicago, Illinois; ^2^Cook County Medical Examiner’s Office, Chicago, Illinois.

In response to the COVID-19 pandemic, Illinois enacted a stay-at-home order on March 21, 2020.* The pandemic caused some persons with opioid use disorder to experience disruptions in treatment and recovery services as well as potential loss of informal social support (*1*). Furthermore, the pandemic has led to interruptions and changes in the illicit drug supply (*1*), which places persons using opioids at increased risk for overdose death. These changes can result in loss of drug tolerance and substitution of powerful illicitly manufactured opioids, such as fentanyl, for less potent drugs that are unavailable during lockdowns (*2*). Finally, persons who had previously used opioids in places where others were present might be alone during a stay-at-home order and therefore at increased risk for fatal overdose, because no bystanders are available to administer naloxone, a medication that can reverse opioid overdose effects when given in time (*1*). Altogether, the challenges for persons with opioid use disorder caused by the COVID-19 pandemic could put them at higher risk for opioid overdose. Increases in overdose deaths during the pandemic have been reported, and detailed recommendations on overdose prevention strategies during COVID-19 have been published (*3*).

Even before the pandemic, an increase in opioid overdose deaths driven by an increasing proportion of fentanyl-related deaths was reported in Illinois and nationwide (*4*,*5*). This report provides estimates of opioid-involved fatal overdoses in Cook County, Illinois (population 5.1 million), which includes the city of Chicago, before, during, and after the Illinois COVID-19 stay-at-home order, which was lifted on May 30.

Data from the Cook County Medical Examiner’s Office Case Archive including deaths with opioid involvement occurring during January 1, 2018–October 6, 2020, were reviewed (*6*). Cause of death determinations are made by forensic pathologists at the Cook County Medical Examiner’s office, which has jurisdiction over all probable drug overdose deaths in Cook County. The cause of death is determined on the basis of autopsy and toxicology findings. The office publishes all the cases it investigates, including cause and manner of death and demographic information, in the Medical Examiner case archive (https://datacatalog.cookcountyil.gov/Public-Safety/Medical-Examiner-Case-Archive/cjeq-bs86), which is updated daily. The number of weekly deaths was calculated for 1) the 99 weeks between January 1, 2018, and December 14, 2019; 2) the 16 weeks before the stay-at-home order was issued (December 15, 2019–March 20, 2020); 3) the 11 weeks during the stay-at-home order (March 21–May 30, 2020); and 4) 18 weeks after the order was lifted (May 31–October 6, 2020). The standard error (SE) of normally distributed period means was used to calculate 95% confidence intervals (CIs) for the mean of weekly deaths in each of the four periods. This research did not involve human subjects.  Research involving deceased persons is not considered human subjects research per the U.S. Department of Health and Human Services Policy for Protection of Human Research Subjects.^†^

A total of 3,843 opioid overdose deaths occurred in Cook County during January 1, 2018–October 6, 2020, with the weekly count of deaths ranging from 12 to 52 (Figure). The weekly mean of 22.6 deaths per week (95% CI = 21.5–23.7) was relatively stable during the initial 99-week period, with little apparent seasonal variation. However, during the subsequent 16 weeks, beginning in December 2019, the mean number of deaths increased to 35.1 per week (95% CI = 32.2–37.8), followed by a more pronounced increase during the 11-week stay-at-home order, with a mean of 43.4 weekly deaths (95% CI = 38.8–48.0). In the 18 weeks after the stay-at-home order was lifted, mean weekly deaths declined to 31.2 (95% CI = 28.6–33.9).

**FIGURE:**
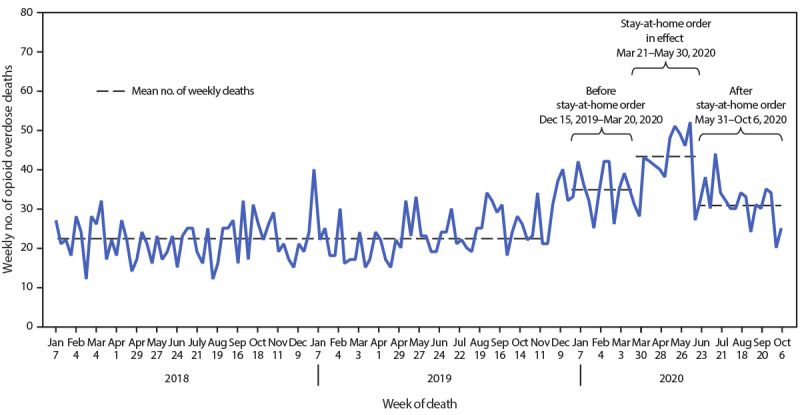
Weekly number of opioid overdose deaths — Cook County, Illinois, January 1, 2018–October 6, 2020

Whether the observed increase during the stay-at-home order was a continuation of increases begun in the 16 weeks before the stay-at-home order or a spike temporally associated with the stay-at-home order is unclear. Although mean deaths have declined below the elevated mean seen during the stay-at-home period, mean opioid overdose deaths in the period after the order was lifted remain elevated above pre-2020 levels. This is concerning because it might indicate an overall persistent upward trend in overdose deaths as reported by CDC, using nationwide data, for the last quarter of 2019 (*5*). As the COVID-19 pandemic continues, outreach, treatment, and recovery organizations have been able to resume some services and initiate others, including online counseling; expanded options for and access to medication-assisted treatment via telehealth; expanded targeted naloxone outreach, education, and distribution in communities with high numbers of overdoses; and creation of online support groups for persons in recovery. These measures might help reduce deaths, especially during another stay-at-home order. Detailed recommendations on overdose prevention strategies during COVID-19 are available (*5*).

## Footnotes

*
https://www2.illinois.gov/pages/executive-orders/executiveorder2020-10.aspx
